# Genome-wide CRISPR/Cas9 library screening identified PHGDH as a critical driver for Sorafenib resistance in HCC

**DOI:** 10.1038/s41467-019-12606-7

**Published:** 2019-10-15

**Authors:** Lai Wei, Derek Lee, Cheuk-Ting Law, Misty Shuo Zhang, Jialing Shen, Don Wai-Ching Chin, Allen Zhang, Felice Ho-Ching Tsang, Ceci Lok-Sze Wong, Irene Oi-Lin Ng, Carmen Chak-Lui Wong, Chun-Ming Wong

**Affiliations:** 1 State Key Laboratory of Liver Research, The University of Hong Kong, Pok Fu Lam, Hong Kong; 2 Department of Pathology, Li Ka Shing Faculty of Medicine, The University of Hong Kong, Pok Fu Lam, Hong Kong

**Keywords:** Cancer, Cancer metabolism, Cancer therapy, Gastrointestinal cancer, Hepatocellular carcinoma

## Abstract

Sorafenib is the standard treatment for advanced hepatocellular carcinoma (HCC). However, the development of drug resistance is common. By using genome-wide CRISPR/Cas9 library screening, we identify phosphoglycerate dehydrogenase (PHGDH), the first committed enzyme in the serine synthesis pathway (SSP), as a critical driver for Sorafenib resistance. Sorafenib treatment activates SSP by inducing PHGDH expression. With RNAi knockdown and CRISPR/Cas9 knockout models, we show that inactivation of PHGDH paralyzes the SSP and reduce the production of αKG, serine, and NADPH. Concomitantly, inactivation of PHGDH elevates ROS level and induces HCC apoptosis upon Sorafenib treatment. More strikingly, treatment of PHGDH inhibitor NCT-503 works synergistically with Sorafenib to abolish HCC growth in vivo. Similar findings are also obtained in other FDA-approved tyrosine kinase inhibitors (TKIs), including Regorafenib or Lenvatinib. In summary, our results demonstrate that targeting PHGDH is an effective approach to overcome TKI drug resistance in HCC.

## Abstract (web summary)

Resistance to the tyrosine kinase inhibitor Sorafenib, which is the standard treatment for advanced hepatocellular carcinoma, is a major clinical challenge. Here, the authors show that phosphoglycerate dehydrogenase, a key enzyme in the serine synthesis pathway, drives sorafenib resistance.

## Introduction

Liver cancer is a common malignancy worldwide and causes more than 700,000 deaths annually^1^. Hepatocellular carcinoma (HCC) is the predominant type of primary liver cancers. HCC is etiologically associated with hepatitis B virus (HBV) and hepatitis C virus (HCV) infection, cirrhosis, alcoholism, and non-alcoholic fatty liver disease (NAFLD). Surgical resection is the mainstay curative treatment for HCC patients. However, due to the aggressive growth and late symptom presentation, most HCC patients are diagnosed at advanced stages and are not eligible for surgical treatments. The median survival of advanced HCC patients is about 9 months and the 5-years overall survival rate is only 10%^2^. Sorafenib, a tyrosine kinase inhibitor (TKI), was the only FDA-approved first-line drug for advanced HCC since 2008, which significantly improved the overall survival of unresectable HCC patients^3^. Recently, Regorafenib, a derivative of Sorafenib, and Nivolumab, an immune checkpoint inhibitor, have been approved by the FDA as second-line treatments for Sorafenib-resistant HCC^4,5^. Most recently, Lenvatinib, another TKI, has shown a comparable survival benefit with Sorafenib in a randomized phase III clinical trial^6^ and has also been approved by FDA as a new first-line treatment for HCC in August 2018. Despite these encouraging advancements, the treatment options for advanced HCC patients remain very limited and further development of new therapeutic regimens is warranted.

Sorafenib targets multiple tyrosine kinases, including RAF, VEGFR, and PDGFR, to suppress their downstream proliferation and survival signaling pathways^7^. However, the clinical efficacy of Sorafenib treatment in HCC is modest and it can only extend patients’ median overall survival by 3 months^3^. The development of drug resistance is considered as a major obstacle contributing to the failure of Sorafenib treatment in HCC patients. Previous studies have shown that Sorafenib treatment restrained tumor growth partly through suppression of tumor angiogenesis. However, tumor hypoxia associated with the Sorafenib treatment can lead to the activation of HIF-1α or HIF-2α in cancer cells, which in turn induces the expression of *VEGF* and other proangiogenic factors to confer HCC resistance to Sorafenib treatment^8,9^. The major mechanism of Sorafenib-mediated anti-proliferative action is through down-regulation of the RAF/MEK/ERK pathway. However, cancer cells can activate alternative signaling pathways, such as EGFR, AKT, and mTOR, to maintain cell proliferation under Sorafenib treatment^10,11^. HCC cells can also elicit autophagy to alleviate ER stress-induced cell death triggered by Sorafenib treatment^12^. Recent studies also reported that Sorafenib treatment could up-regulate the expression of stem cells markers CD44 and CD47 and enrich the liver cancer stem cell populations in the tumor. Liver cancer stem cells are refractory to Sorafenib and may therefore account for the tumor remission after prolonged Sorafenib treatment in HCC patients^13,14^. Nevertheless, due to the tolerable safety profile and manageable side effects, Sorafenib is an attractive molecular targeted drug in the clinical setting. To overcome Sorafenib resistance, it is increasingly favorable to develop a combinational therapy with other anti-cancer drugs, especially those targeting molecules involved in Sorafenib resistance. For instance, co-treatment of EGFR inhibitor Gefitinib or anti-CD47 antibody could effectively improve the anti-cancer effect of Sorafenib in the mouse models^10,13^. The underlying mechanisms of Sorafenib resistance are complicated and remain largely elusive. Further investigations on the molecular basis of Sorafenib resistance may shed light on the identification of new targets for rational combinational therapy to overcome Sorafenib resistance.

High-throughput forward genetic screening approaches have been widely applied to study the molecular mechanisms associated with specific cellular phenotypes, including drug resistance in human cancers. RNAi screening using shRNA library to down-regulate specific target genes is a well-established method for loss-of-function screening. Previous pooled shRNA library screening in HCC-bearing mouse has identified MAPK14 as a critical player involved in Sorafenib resistance^15^. However, RNAi-based screening has some limitations. RNAi only knocks down the target mRNA expression but not completely eradicate the target gene. The inefficient gene knockdown results in residual mRNA expression that may obscure the observation of the loss-of-function phenotype, thereby leading to false-negative results. Another major challenge is the prevalent off-target effects that may inadvertently perturb the expression of other off-target genes, causing false-positive results^16^. Recent innovations in genome editing technology especially the CRISPR/Cas9 system have hugely accelerated the functional genomic researches in mammalian cells. The CRISPR/Cas9 system was first discovered in bacteria and archaea as an adaptive immune mechanism to protect from viral DNA invasion^17^. In mammalian cells, the CRISPR/Cas9 system has been engineered to introduce frameshift mutation for specific gene knockout. Because of the easy programmability and high gene-editing efficacy, the CRISPR/Cas9 system has been increasingly applied to study loss of gene functions in a variety of biological systems. Recently, different CRISPR/Cas9 libraries have been developed for genetic screening in mammalian cell culture and mouse models^18–20^. The CRISPR/Cas9 library screens have been utilized to identify genes that are important for cancer cell survival, proliferation, migration, and resistance to drug treatment in various models^19,20^. Compared with previous RNAi-based loss-of-function screening, CRISPR/Cas9 knockout library provides a higher screening sensitivity, since incomplete knockdown by RNAi sometimes may not be sufficient to generate the loss-of-function phenotype. Moreover, CRISPR/Cas9 library screening also outperforms RNAi screening with lower noise, minimal off-target effects and higher data reproducibility^21^.

In this study, we perform a genome-wide CRISPR/Cas9 knockout screening in HCC cells with Sorafenib and vehicle control treatments to systematically evaluate the driving mechanisms of Sorafenib resistance. We identify phosphoglycerate dehydrogenase (PHGDH), the key enzyme in serine synthesis pathway (SSP), as a critical driver of Sorafenib resistance in HCC. Sorafenib treatment elevates cellular ROS level and induces cell apoptosis. HCC cells activate PHGDH and serine synthesis pathway to generate antioxidant and α-ketoglutarate to survive Sorafenib -induced oxidative stress. Inactivation of PHGDH sensitizes HCC to Sorafenib-induced cell apoptosis. Treatment of PHGDH inhibitor NCT-503 acts synergistically with Sorafenib to suppress HCC cell growth in the mouse model. Interestingly, PHGDH also involves in drug resistance to Regorafenib and Lenvatinib. Our findings indicate that targeting PHGDH is a promising strategy to overcome TKI drug resistance in human HCC.

## Results

### CRISPR library screening identified PHGDH as a critical gene for Sorafenib resistance

**Fig. 1:**
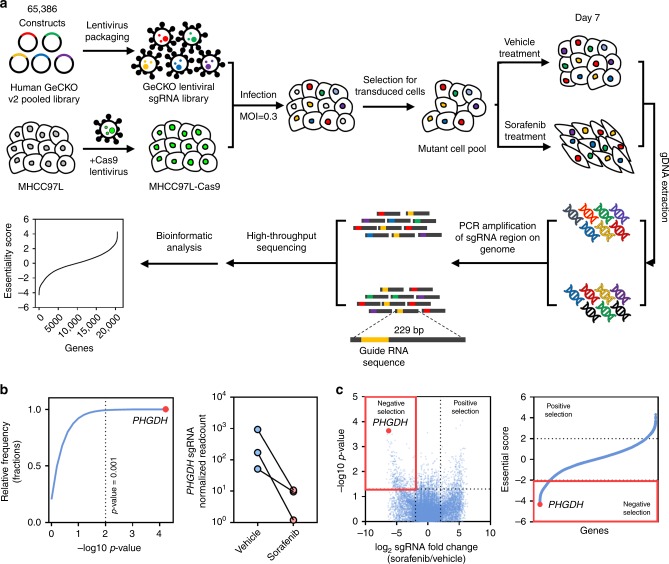
CRISPR library screening identified PHGDH as a driver for Sorafenib resistance. **a** Schematic diagram illustrates the workflow of genome-wide CRISPR/Cas9 knockout library screening (CRISPR: Clustered Regularly Interspaced Short Palindromic Repeats). Human genome-wide CRISPR/Cas9 knockout library (GeCKO v2A) containing 65,386 sgRNAs was packed into lentiviral particle and transduced into Cas9-overexpressing MHCC97L cells (MHCC97L-Cas9) at low multiplicity of infection (MOI). The sgRNA transduced cells were selected by puromycin to generate a mutant cell pool. Mutant cells were cultured in vehicle and Sorafenib for 7 days for genetic screening. Genomic DNA was extracted from the treated cells and the sgRNA fragment was amplified by PCR. Copy number of sgRNAs was determined by high-throughput sequencing and analyzed by MAGeCK v0.5.7 algorithm. **b** *PHGDH* (phosphoglycerate dehydrogenase) was identified as the most significant gene in the library screening as indicated by the red dot. The sgRNAs targeting *PHGDH* were consistently depleted in Sorafenib-treated cells. **c** Volcano plots revealed that *PHGDH* targeting sgRNAs were negatively selected during Sorafenib treatment, suggesting that *PHGDH* is an essential gene for HCC cells to survive from Sorafenib treatment. Source data are provided as a Source Data file

**Fig. 2:**
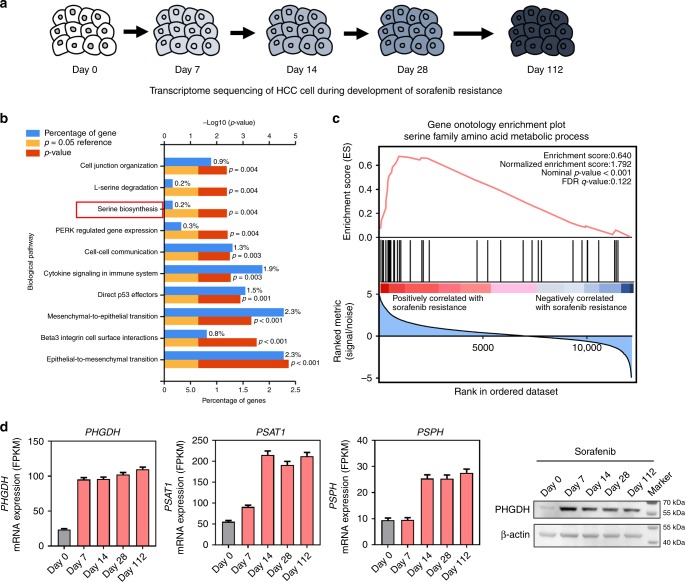
Activation of serine synthesis pathway was involved in Sorafenib resistance. **a** MHCC97L cells were treated with Sorafenib for 112 days. RNA-Seq was performed in Sorafenib treated MHCC97L cells at the indicated time points. **b** DAVID gene ontology and **c** Gene Set Enrichment Analysis (GSEA) showed that genes involved in serine biosynthesis were significantly deregulated in Sorafenib-resistant cells (Day 112) when compare to the parental cells (Day 0). **d** RNA-Seq analysis revealed that the key enzymes in serine synthesis pathway, PHGDH, PSAT1 (phosphoserine aminotransferase 1), and PSPH (phosphoserine phosphatase), were all up-regulated in Sorafenib-resistant cells. The upregulation of PHGDH was also confirmed in protein level by western blot. The error bar represents the mean with upper and lower limit. Source data are provided as a Source Data file

In this study, we performed genome-wide CRISPR/Cas9 knockout library screening to identify critical genes involved in Sorafenib resistance in human HCC. The HCC cell line MHCC97L which has relatively higher GI50 against Sorafenib was used as the in vitro model to overexpress Cas9 protein (Fig. S1a). Human GeCKO v2 CRISPR library A contains 65,386 unique sgRNAs, which targets 19,052 protein-coding genes, and 1864 microRNAs were used to generate a mutant cell pool. We then treated the mutant cell pool with vehicle or Sorafenib for 7 days to enable positive and negative screening (Fig. 1a). We hypothesized that knockout of a Sorafenib resistance driver gene will sensitize HCC cells to Sorafenib-induced cell death or proliferation suppression. In the presence of Sorafenib, cells carrying sgRNA targeting Sorafenib resistance genes will be negatively selected in the mutant cell pool and their corresponding sgRNA will also be depleted in the library that can be determined by high-throughput sequencing. We found that 7 μM of Sorafenib treatment effectively suppressed cell proliferation compared with the vehicle-treated group (Fig. S1b), which suggested an effective selection pressure. After selection, we achieved about 400× coverage of the library and around 94% of the sgRNA sequences were retained in all samples, which ensure the sufficient read-death and library coverage for the CRISPR library screening (Fig. S1c). From this CRISPR/Cas9 knockout library screening, we identified a subset of sgRNAs targeting 984 genes were significantly depleted (*P* < 0.05) in the Sorafenib-treated cells when compared to vehicle control, indicating that these genes might be potential drivers for Sorafenib resistance (Fig. S1d). Pathway analysis (DAVID Bioinformatics Resources 6.8) suggested that these genes were involved in cell growth and adhesion, protein tyrosine kinase activity, and regulation of MAPK cascade, which echoed previous pooled shRNA library screening results (Fig. S1e)^15^. Among the list of genes, phosphoglycerate dehydrogenase (PHGDH), the rate-limiting enzyme for SSP, was identified as the most negatively selected gene upon Sorafenib treatment. All *PHGDH* targeting sgRNAs were dramatically decreased in Sorafenib-treated cells, implying that loss of PHGDH might sensitize HCC cells to Sorafenib treatment (Fig. 1b, c). In addition, we tested the effects of other genes on the top of the gene list identified by CRISPR/Cas9 knockout library screening. We found that knockdown of AKT1S1, TBL1Y, SKAP2, and AMPD2 significantly induced apoptosis in HCC cells only in the presence of Sorafenib, suggesting these genes were also involved in Sorafenib resistance and the result from CRISPR/Cas9 knockout library screening could be recapitulated (Fig. S2a). In parallel with the CRISPR/Cas9 knockout library screening, we also performed RNA-seq to monitor the transcriptomic changes of MHCC97L cells under Sorafenib treatment for up to 4 months (Fig. 2a). The successful development of Sorafenib-resistant cells was evidenced by the increased cell viability and reduced apoptosis under Sorafenib treatment (Fig. S2c, d). Pathway analysis (DAVID 6.8 and GSEA) suggested that activation of SSP was found in Sorafenib-resistant cells (Fig. 2b, c). In particular, the expressions of key SSP enzymes, PHGDH, phosphoserine aminotransferase 1 (PSAT1), and phosphoserine phosphatase (PSPH), were all induced by Sorafenib treatment (Fig. 2d). The induction was further validated using qRT-PCR (Fig. S2e). The above results indicated that PHGDH and SSP may play an important role in the development of Sorafenib resistance.

### Inhibition of PHGDH sensitized HCC cells to Sorafenib treatment

**Fig. 3:**
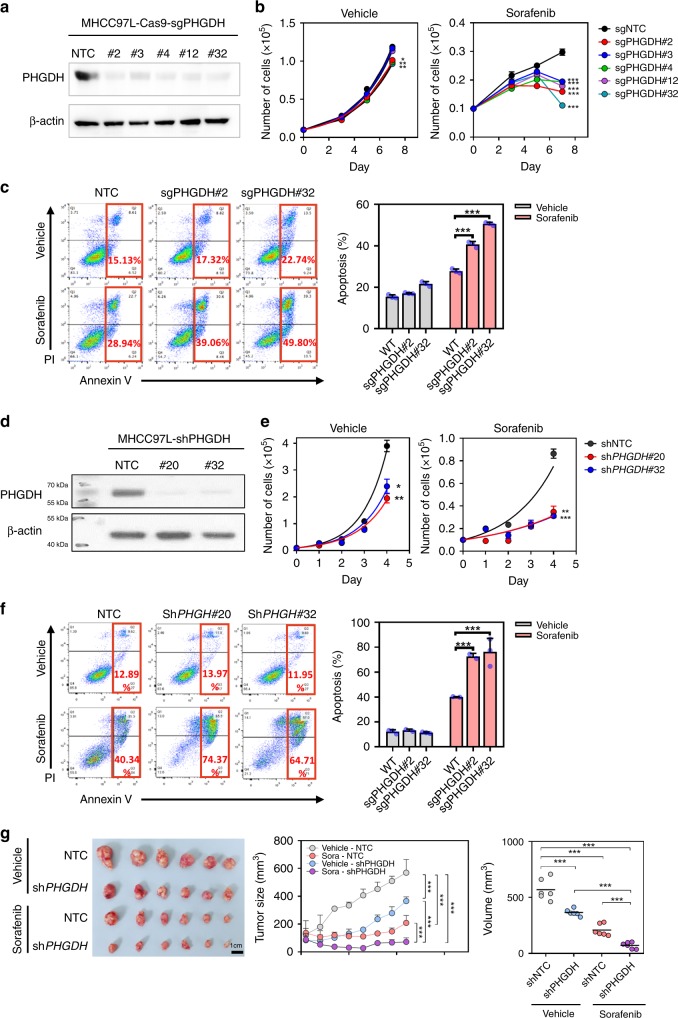
Depletion of *PHGDH-*sensitized HCC cells to Sorafenib treatment. **a** Knockout of *PHGDH* in MHCC97L cells by CRISPR/Cas9 gene editing system. **b** Knockout of *PHGDH* showed mild effect on cell proliferation in in vitro cell culture. However, knockout of *PHGDH* significantly suppressed MHCC97L cell proliferation in the presence of Sorafenib (black connected dots: non-target control; red connected dots: sgPHGDH#2; deep blue connected dots: sgPHGDH#3; green connected dots: sgPHGDH#4; purple connected dots: sgPHGDH#12; light blue connected dots: sgPHGDH#32). **c** Knockout of *PHGDH* induced apoptosis significantly upon Sorafenib treatment (gray bar: vehicle-treated group; red bar: Sorafenib-treated group). **d** Knockdown of *PHGDH* by lentiviral-based shRNA approach. **e** Knockdown of *PHGDH* suppressed HCC cell proliferation under Sorafenib treatment (black connected dots: non-target control; red connected dots: shPHGDH#20; deep blue connected dots: shPHGDH#32). **f** Knockdown of *PHGDH* augmented Sorafenib induced apoptosis in HCC cells (gray bar: vehicle-treated group; red bar: Sorafenib-treated group). **g** Knockdown of *PHGDH* sensitized HCC cell to Sorafenib in nude mice (gray connected dots: Vehicle-NTC, non-target control treated with vehicle; blue connected dots: Sora-NTC, non-target control treated with Sorafenib; red connected dots: Vehicle-shPHGDH, *PHGDH* knockdown clones treated with vehicle; purple connected dots: Sora-shPHGDH, *PHGDH* knockdown clones treated with Sorafenib). The error bar in panels **b**, **c**, **d**, **f** represents the standard error of mean (SEM), *n* = 3 biologically independent samples. The error bar in panel **g** represents the standard deviation (SD), *n* = 6 mice. Source data are provided as a Source Data file. (Student's *t*-test *P < 0.05, **P < 0.01, ***P < 0.001)

To validate the results from our CRISPR/Cas9 knockout library screening, we generated PHGDH stable knockout (KO) subclones by infecting MHCC97L-Cas9 cells with the three sgRNAs included in the library and two additional independent sgRNAs. Western blotting confirmed that all sgRNA sequences were able to effectively knock out PHGDH in MHCC97L cells (Fig. 3a). PHGDH KO subclones showed mild suppressive effects on HCC cell proliferation in vitro, whereas KO of PHGDH effectively impeded cell proliferation (Fig. 3b) and significantly induced apoptosis (Fig. 3c) in the presence of Sorafenib. To consolidate our CRISPR/Cas9 knockout library screening result with an orthogonal approach, we generated PHGDH knockdown subclones in MHCC97L using two independent shRNA sequences (Fig. 3d). Consistently, shRNA-mediated knockdown of PHGDH recapitulated the sensitization effect of sgRNA-mediated KO in HCC cells towards Sorafenib treatment (Fig. 3e). PHGDH knockdown also dramatically induced apoptosis in the presence of Sorafenib (Fig. 3f). Next, we sought to confirm this observation using an in vivo model. We inoculated the PHGDH knockdown (shPHGDH) and the non-target control (NTC) MHCC97L in the left and right flanks of nude mice, respectively. When tumors became palpable at day 21, mice were treated with Sorafenib (Sora) or vehicle control (Vehicle) for another 21 days. More remarkably, while tumors raised from NTC cells grew steadily, Sorafenib treatment completely retarded tumor growth of PHGDH cells in mice (Fig. 3g). The above findings suggested that inactivation of PHGDH sensitize HCC cells to Sorafenib treatment.

### PHGDH increased αKG and NADPH production

**Fig. 4:**
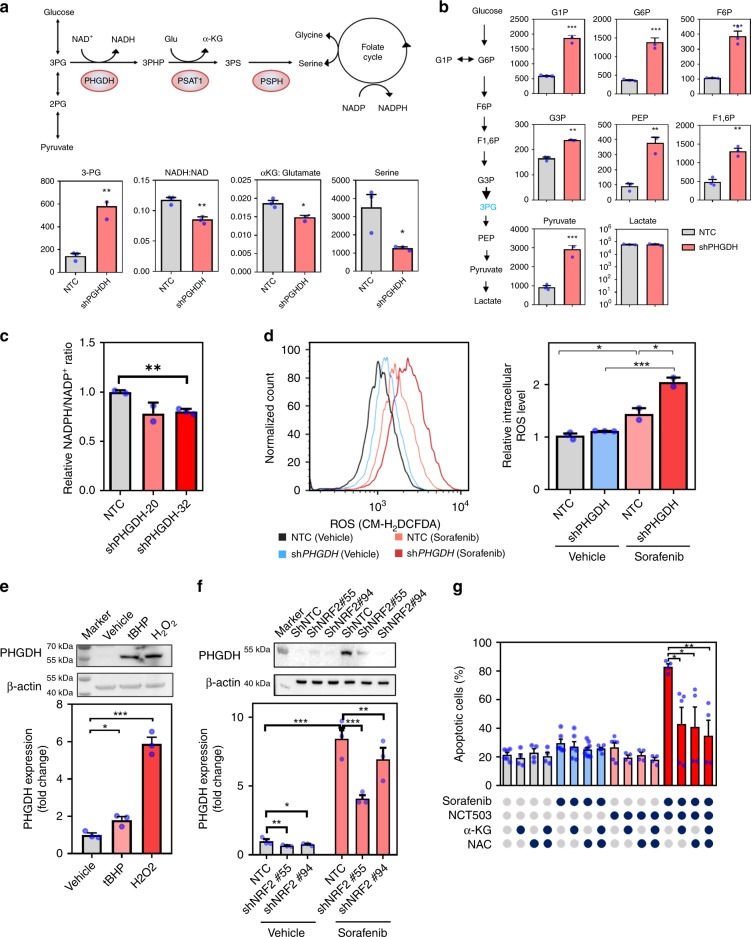
Knockdown of *PHGDH* impaired serine biosynthesis and induced oxidative stress. **a** Knockdown of *PHGDH* impaired serine synthesis pathway (SSP), leading to accumulation of 3-phosphoglyceric acid (3-PG) and reduced production of nicotinamide adenine dinucleotide+hydrogen (NADH), α-ketoglutarate (α-KG), and serine. **b** Glycolytic metabolites cannot be branched into SSP upon knockdown of *PHGDH*, causing the accumulation of most glycolytic metabolites glucose 1-phosphate (G1P), glucose-6-phosphate (G6P), fructose 6-phosphate (F6P), fructose 1,6-bisphosphate (F1,6P), glyceraldehyde 3-phosphate (G3P), phosphoenolpyruvate (PEP), and pyruvate (gray bar: non-target control; red bar: *PHGDH* knockdown clones). **c** Knockdown of *PHGDH* resulted in reduced nicotinamide adenine dinucleotide phosphate (NADPH) production. **d** Sorafenib treatment augmented reactive oxygen species (ROS) level. Knockdown of *PHGDH* intensified the Sorafenib-induced oxidative stress in HCC cells (black line: non-target control treated with vehicle; light red line: non-target control treated with Sorafenib; blue line: *PHGDH* knockdown clones treated with vehicle; deep red line: *PHGDH* knockdown clones treated with Sorafenib). **e** H_2_O_2_ and *tert*-butyl hydroperoxide (tBHP) treatment at 20 and 200 μM for 24 h respectively induced PHGDH expression in HCC cells in both mRNA and protein level. **f** Knockdown of NRF2 (Nuclear factor erythroid 2-related factor 2) alleviated Sorafenib-induced PHGDH up-regulation in HCC cells in both mRNA and protein level. **g** Treatment of α-KG at 4 mM and NAC at 5 mM for 48 h substantially inhibited Sorafenib (5 μM) and NCT-503(40 μM)-induced apoptosis in HCC cells. The error bar represents SEM. *n* = 3 biologically independent samples in panels **a**–**f** and 5 in panel **g**. Source data are provided as a Source Data file (Student's *t*-test *P < 0.05, **P < 0.01, ***P < 0.001)

To confirm the effect of PHGDH on Sorafenib resistance, we subjected the knockdown clone with the best knockdown efficiency to metabolomics study by LC-MS analysis. Knockdown of PHGDH caused accumulation of 3PG and significantly reduced the ratio of NADH to NAD, αKG to glutamate, and serine, suggesting a decrease of metabolites entering the SSP (Fig. 4a). Glycolytic metabolites are channeled to SSP through PHGDH. Knockdown of *PHGDH* concomitantly caused an accumulation of most glycolytic metabolites G1P, G6P, F6P, F1,6P, G3P, PEP, and pyruvate, suggesting that glycolytic metabolites cannot be branched into SSP upon knockdown of *PHGDH*. Interestingly, the level of lactate did not change, indicating that the overall glucose uptake rate may not be altered (Fig. 4b). To directly evaluate the effects of Sorafenib on serine synthesis, we cultured MHCC97L cells with [U-13C]-glucose with Sorafenib and vehicle, respectively, for 48 h to study the level of C13 labeled serine by mass spectrometry. Sorafenib significantly increased serine (M+3) in HCC cells (Fig. S3). Serine is connected with the folate cycle to generate important antioxidant NAPDH. As NADPH is a relatively unstable metabolite, we quantitated the level of NADPH independently using NADPH detection kit. Knockdown of *PHGDH* profoundly reduced NADPH production (Fig. 4c). As NADPH is the key antioxidant which maintains the reducing power of thioredoxin and glutathione, we next examined the level of oxidative stress by a general reactive oxygen species (ROS) dye (CMH_2_DCFDA) and a mitochondrial ROS dye, MitoSOX^TM^. Knockdown of *PHGDH* or Sorafenib treatment independently elevated ROS level. Knockdown of *PHGDH* drastically induced ROS in the presence of Sorafenib (Fig. 4d and S4a). Interestingly, scanning electron microscopy showed that cristae of the mitochondria were less intact after single drug treatments and were further exacerbated with combined treatment (Fig. S4b). In addition, we showed that *PHGDH* expression was remarkably elevated upon oxidative stress induced by H_2_O_2_ or tBHP treatment (Fig. 4e). We further demonstrated that the PHGDH induction was dependent on NRF2, the master transcriptional regulator of the oxidative stress response, as *NRF2* was significantly induced by Sorafenib treatment (Fig. S5a) and knockdown of *NRF2* significantly reduced the mRNA and protein expression of PHGDH in the presence of Sorafenib (Fig. 4f). Previous study in non-small lung cancer suggested that NRF2 regulate PHGDH expression through ATF4 (ref. ^22^). To confirm whether ATF4 is involved in regulating PHGDH in HCC, we established the *ATF4* knockdown MHCC97L cell line and validated the successful knockdown of *ATF4* in the cells (Fig. S5b). Interestingly, we found that *ATF4* can also be induced by Sorafenib treatment (Fig. S5b), and the expression of *PHGDH* upon Sorafenib treatment was significantly decreased in ATF4 stable knockdown cells in both mRNA (Fig. S5c) and protein level (Figure S5d). The result indicated that ATF4 was also involved in regulating *PHGDH* expression in HCC cells. More intriguingly, cell-permeable-αKG or antioxidant *N*-acetyl cysteine (NAC) could partially rescue the effect inflicted by combined treatment of Sorafenib and PHGDH inhibitor (NCT-503), suggesting that PHGDH inhibitor sensitized HCC cells to Sorafenib through depleting αKG and increasing ROS production (Fig. 4g). This observation echoes with the metabolic changes induced by *PHGDH* knockdown. The above findings also suggested that HCC cells cope with the oxidative stress induced by Sorafenib treatment by increasing the expression of *PHGDH*. Inactivation of PHGDH reduced the REDOX capacity, therefore making the cells more vulnerable to Sorafenib.

### PHGDH inhibitor sensitized HCC cells to Sorafenib treatment

**Fig. 5:**
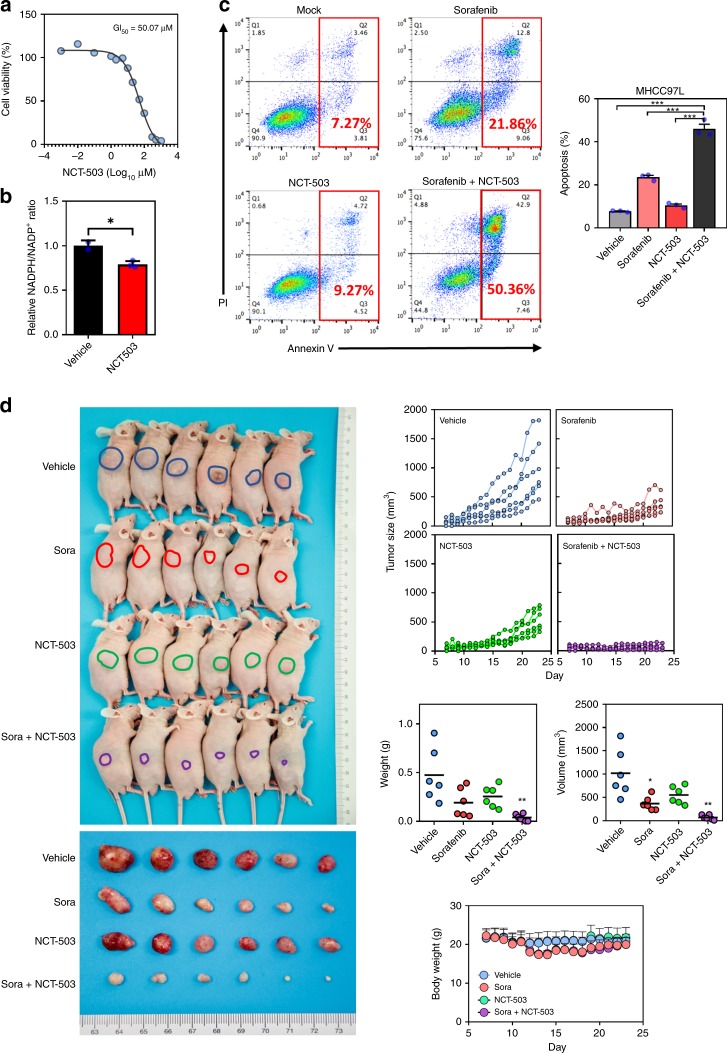
PHGDH inhibitor sensitized HCC cells to Sorafenib treatment. **a** NCT-503 inhibited HCC cell proliferation in a dose-dependent manner with 50% growth inhibiting (GI50) at 50 µM. **b** Treatment of NCT-503 at 40 μM for 48 h significantly reduced the relative ratio of NAPDH/NAD+in HCC cells. **c** Annexin V-PI staining showed that NCT-503 worked synergistically with Sorafenib to induce HCC cell apoptosis. **d** Both NCT-503 and Sorafenib inhibited HCC tumorigenicity. NCT-503 and Sorafenib combination therapy effectively abolished HCC growth in the nude mice model (blue connected dots: vehicle-treated group; red connected dots: Sorafenib-treated group; green connected dots: NCT-503-treated group; purple connected dots: Sorafenib and NCT-503-treated group). The error bar in panels **b** and **c** represents the SEM, *n* = 3 biological independent samples. The error bar in panel **d** represents the SD, *n* = 6 mice. Source data are provided as a Source Data file (Student's *t*-test *P < 0.05, **P < 0.01, ***P < 0.001)

NCT-503 is a small molecular inhibitor specific for PHGDH inhibition. We determined the GI50 of NCT-503 in the MHCC97L cell line (Fig. 5a) and found that treatment of NCT-503 significantly reduced the relative ratio of NADPH/NADP+ in cells (Fig. 5b). To test whether PHGDH inhibitor could work synergistically with Sorafenib in suppressing HCC growth, we treated MHCC97L cells with 4 µM Sorafenib alone, 40 µM NCT-503 alone, and 4 µM Sorafenib, and 40 µM NCT-503 together for 48 h. Annexin V and PI staining demonstrated that Sorafenib alone could induce apoptosis. Strikingly, NCT-503 could double the number of the apoptotic cells induced by Sorafenib despite NCT-503 itself could only slightly induce apoptosis (Fig. 5c). To test whether the synergistic effect of NCT-503 with Sorafenib was specifically due to PHGDH inhibition, we also tested other PHGDH inhibitors, NCT-502 and CBR-5884 (Fig. S6a). Consistently, both could significantly elevate apoptotic cell population induced by Sorafenib (Fig. S6b). In the Sorafenib-resistant MHCC97L cells we established earlier, we found that apoptosis was significantly reduced as compared to parental HCC cells under Sorafenib single treatment. However, serine inhibition through NCT-503 successfully induced apoptosis of Sorafenib-resistant MHCC97L cells together with Sorafenib, further suggesting that NCT-503 effectively overcame Sorafenib resistance (Fig. S2d). Next, we performed subcutaneous injection of MHCC97L cells in nude mice. When tumors were palpable, mice were divided into four groups randomly and were treated with Sorafenib alone, NCT-503 alone, Sorafenib and NCT-503, and vehicle controls. Sorafenib and NCT-503 were administered orally and intraperitoneally, respectively. Sorafenib and NCT-503 alone could reduce HCC growth in vivo while combined treatment of Sorafenib and NCT-503 completely halted HCC growth in vivo (Fig. 5d). Of note, the body weights of mice in the three drug-treated groups remained unchanged (Fig. 5d). Also, there was no observable abnormal behavior in mice during the course of drug treatment. These data together confirmed that PHGDH inhibitor could sensitize HCC to Sorafenib treatment.

### Activation of SSP is a general mechanism for TKI resistance

**Fig. 6:**
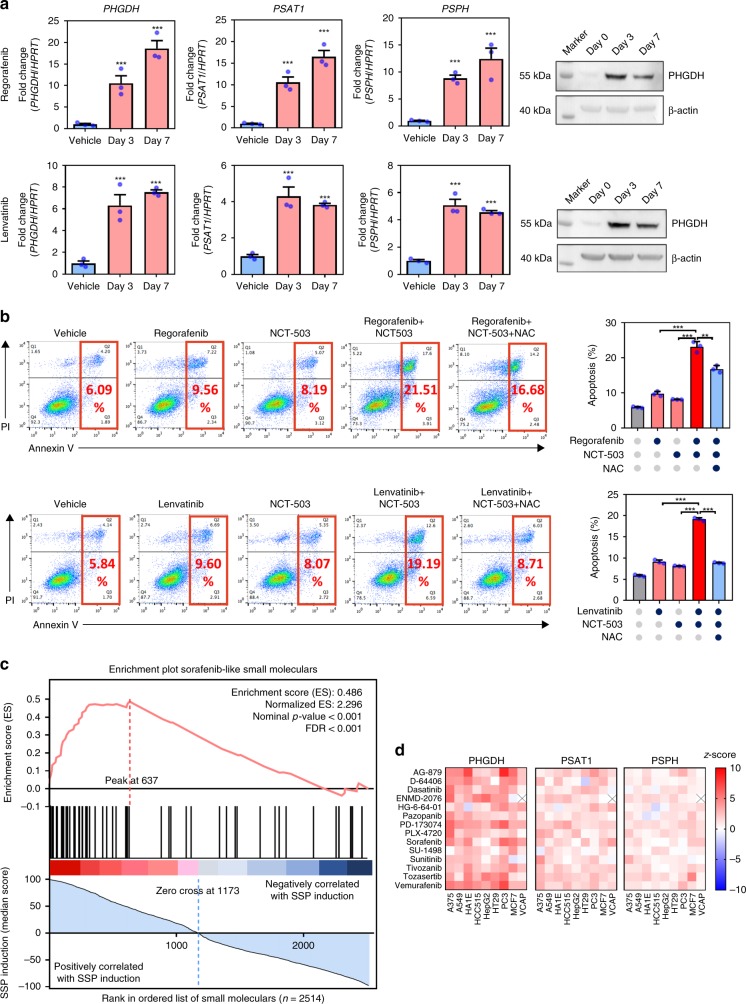
PHGDH contributed to Regorafenib and Lenvatinib resistances in HCC. **a** Treatment of Regorafenib at 10 μM and Lenvatinib at 40 μM for 48 h induced *PHGDH*, *PSAT1*, and *PSPH* expression in HCC cells. **b** Co-treatment of NCT-503 intensified Regorafenib and Lenvatinib-induced apoptosis in HCC cells, which can be rescued by addition of 5 mM NAC of 48 h treatment. **c** In total, 2514 compounds are ranked by their ability of inducing SSP in nine cell lines. The Sorafenib-like small molecules (*n* = 52) were significantly enriched in the highly ranked SSP-inducible compounds as suggested by GSEA pre-ranked enrichment analysis. **d** Most of the Sorafenib-like small molecules profoundly induced the mRNA expression of *PHGDH*, *PSAT1*, and *PSPH* in all cell lines. The error bar represents the SEM, *n* = 3 biological independent samples. Source data are provided as a Source Data file. (Student's *t*-test *P < 0.05, **P < 0.01, ***P < 0.001)

To confirm that our study was not limited to Sorafenib, we treated MHCC97L with two other TKIs, Regorafenib and Lenvatinib. Our findings showed that Regorafenib and Lenvatinib could both induce *PHGDH*, *PSAT1*, and *PSPH* expressions as early as 3 days after treatment (Fig. 6a). Similar to Sorafenib, Regorafenib and Lenvatinib could slightly induce apoptosis. Strikingly, NCT-503 worked together with Regorafenib or Lenvatinib to profoundly increase apoptosis in HCC cells (Fig. 6b). These findings suggested that elevation of SSP by up-regulation of *PHGDH*, *PSAT1*, and *PSPH* is a common mechanism underlying TKI resistance in HCC and thus targeting PHGDH may be a promising combinational therapy strategy to improve the efficacy of TKI treatment in HCC patient. To investigate whether induction of SSP could be a signature of other Sorafenib-like small molecules in the treatment of different types of cancers, we examined the transcriptional expression profiles of 2514 small molecules available on Connectivity Map (CMap, Broad Institute)^23^ and ranked them by their ability of inducing SSP (Fig. 6c). To our surprise, we found that the small molecules (*n* = 52) in the same pharmacology class with Sorafenib (RAF inhibitors, VEGFR inhibitors, PDGFR receptor inhibitors, FLT3 inhibitors, KIT inhibitors, RET tyrosine kinase inhibitors) are significantly enriched in the highly ranked SSP-inducing compounds (Fig. 6c). These pharmacology classes are also on the top of the list for inducing SPP among all 171 classes on CMap (Fig. S7). Most of the Sorafenib-like small molecules (Table S1) could significantly induce the expression of *PHGDH*, *PSAT1*, and *PSPH* across various types of cancer cells (Fig. 6d), suggesting that induction of SSP is a common event in response to Sorafenib-like small molecules treatment and targeting PHGDH may be applied in a wider range of combinational treatment for a more variety of cancer types.

## Discussion

From an unbiased genome-wide CRISPR/Cas9 KO library screening coupled with transcriptome sequencing, we identified the SSP as the most important pathway responsible for TKI resistance in HCC and we demonstrated that PHGDH inhibitor targeting the first enzyme of SSP sensitized HCC cells to TKI treatment.

CRISPR/Cas9 knockout library screening enables researchers to identify genes contributed to a specific phenotype (i.e. a forward genetic screening) in a genome-wide scale. In this study, we adopted the library screening strategy using the GeCKO v2A library as previously described by others^24–26^. The quality check of our library screening was conducted by accessing the read depth, the number of missing sgRNAs, sgRNA coverage in each group. Moreover, we found that the previously reported 1580 essential genes^27,28^ were significantly depleted at day 7 of the vehicle control group as compared to day 0, thus confirmed the reliability of the screening (Fig. S2b).

Functionally, we validated the effect of PHGDH in Sorafenib resistance by three sgRNAs in the library and two additional sgRNAs. In addition to PHGDH, we also confirmed the effects of top genes (AKT1S1, TBL1Y, SKAP2, and AMPD2) from the screening on Sorafenib resistance (Fig. S2a). Our knockout library screening result also echoed with previous pooled shRNA knockdown library conducted in mouse HCC model which identified MAPK pathway as an important mechanism of Sorafenib resistance in HCC (Fig. S1e)^15^.

Most studies in the past on serine and SSP hinged on their functions in neurobiology. Serine is well-known to be a neurotransmitter and *PHGDH* KO mice exhibited neurological disorder and could not survive long after birth^29,30^. The SSP branching from glycolysis consists of three committed steps which are carried out by three enzymes, PHGDH, PSAT1, and PSPH. PHGDH carries out an NAD^+^ dependent oxidation of 3PG to 3PHP. PSAT1 carries out the transamination reaction converting 3PHP to 3PS using glutamate as an amino donor producing αKG. PSPH finally dephosphorylates 3PS into serine. Serine is a metabolite closely connected to the folate cycle in generating antioxidant. SHMT converts serine to glycine, donating 1-carbon to the tetrahydrofolate backbone to initiate the folate cycle. Enzymes in the folate cycle including MTHFD1, MTHFD2/L, and ALDH1L1/2 directly produce the major antioxidant NADPH. Folate cycle also generates 5,10-meTHF which is used for thymidylate production by TYMS and methionine production by MTHFR and MTR. Folate cycle also generates 10-formyl THF which is used for purine production by GART/ATIC. In sum, the SSP coupled with the folate cycle together generates several key metabolites including NADH, αKG, serine, NADPH, thymidylate, methionine, purines, and serine. Serine is important for phosphatidyl-serine and sphingosine production. Serine also serves as the precursor of glycine and cysteine which are two core components of glutathione^31^. As excessive serine (around 0.4 mM) is present in our culture medium, serine per se is not sufficient for conferring HCC cells TKI resistance given the serine transporters in HCC cells are expressed. Our data suggested that exogenous serine could not replace de novo generation of serine through SSP due to the additional metabolites produced by SSP.

Independent groups have demonstrated the different pro-tumorigenic effects of PHGDH in the breast cancer cell model^32,33^. Possemato et al.^33^ showed that knockdown of *PHGDH* and *PSAT1* reduced a significant proportion of cellular αKG which supports the TCA cycle for anaplerotic reactions. Meanwhile, Pacold et al.^34^ demonstrated that inhibition of PHGDH by NCT-503 repressed the production of thymidylate and AMP. Here, we showed that αKG and NAC could rescue apoptosis in Sorafenib-treated HCC cells, suggesting that the sensitization effect of PHGDH inhibition was associated with depletion of αKG and augmentation of ROS. αKG replenishes TCA cycle for anaplerotic reactions. αKG is required for enzymatic reactions carried out by demethylases (TET and KDM) and prolyl hydroxylases (PHDs). The decrease of αKG could result in hypermethylation in DNA and histone, causing a change in the epigenetic landscape. The decrease of αKG could result in activation of HIFs which are counteracted by PHDs. Isotope tracing experiment demonstrated that PHGDH contributed to at least 50% of αKG pool in the cells. Strikingly, we found that αKG markedly prevented apoptosis under Sorafenib treatment, highlighting the roles of PHGDH might be beyond its metabolic functions.

Genomic amplification of PHGDH at chromosome 1p was found to be the cause of its over-expression in melanoma and breast cancer. Over-expression of *PHGDH* could also be driven by the activation of transcription factors. Furthermore, *PHGDH*, *PSAT1*, *PSPH*, and *SHMT* have been shown to be direct transcriptional targets of ATF4 which was transcriptionally activated by NRF2 in a non-small-cell lung cancer model^22^. Up-regulation of *PHGDH* was associated with poor prognosis in breast cancer patients^35,36^. Transcription of *PHGDH*, *PSAT1*, *PSPH* could be activated by c-MYC under nutrient starvation such as glucose, glutamine, or serine/glycine free conditions. c-MYC-driven SSP promoted HCC survival through nucleotide and GSH synthesis, enabling cells to counteract ROS, evade apoptosis, and undergo cell cycle progression^37^. Consistent with another report, we found that *PSPH* was the only SSP gene that was over-expressed in human HCC while *PHGDH* and *PSAT1* were controversially under-expressed in human HCC^37^. However, TKIs could clearly induce all SSP genes, suggesting that SSP provides unique advantages for HCC cells survival in TKI-induced stress. As SSP genes were induced shortly after TKI treatment, we speculate that this response is an adaptive response mediated by transcription factors rather than genetic alterations.

NCT-503 was identified as a specific PHGDH inhibitor from NIH Molecular Libraries Small Molecule Repository (MLSMR) drug library containing 40,000 compounds^34^. Using same screening approach which involves PHGDH activity assay coupled with diaphorase enzyme which converts resazurin to resorufin as the readout, another group identified another PHGDH inhibitor, CBR-5884, from a small-molecule drug library containing 800,000 compounds^38^. Although CBR-5884 has low solubility in vivo, CBR-5884 demonstrated great effects in SSP and growth inhibition in breast and melanoma cells in vitro^38^. Both NCT-503 and CBR-5884 displayed higher growth inhibitory effects in cancer cells which express a high level of PHGDH especially under extracellular serine-depleted condition^34,38^. Future studies which involve the modification of PHGDH inhibitors to increase its bioavailability will popularize the usage of PHGDH inhibitors as a cancer therapy.

Our study was the first which employed unbiased whole-genome CRISPR-library screening to systematically identify *PHGDH* and the SSP as the most significant gene and pathway associated with TKI resistance. This is especially significant for liver cancer treatment as TKI represents the only first-line treatment option for HCC patients. Scattered studies have briefly reported the importance of PHGDH in drug resistance. PHGDH was found to be associated with the resistance of Bortezomib in myeloma cells using metabolomics and proteomics approaches^39^. PGHDH was also found to be associated with Sunitinib resistance in renal cancer using transcriptome sequencing approach^40^. Although the mechanisms of action of these drugs vary, these data together with ours corroborate the importance of metabolic rewiring in survival adaption under drug treatment. SSP represents a metabolic vulnerability of cancer cells under drug treatment while targeting SSP through PHGDH inhibitors is an attractive therapeutic approach for TKI resistant HCC.

## Methods

### Genome-wide CRISPR/Cas9 knockout library screen

In this study, the Human GeCKOv2A CRISPR knockout pooled library was used to identify genes responsible for Sorafenib resistance in HCC cells. The library was a gift from Feng Zhang (Addgene # 1000000049)^41^. The workflow of this forward genetic screen is illustrated in Fig. 1a. First, we established a stable Cas9-expressing HCC cell line (MHCC97L-Cas9) by lentiviral transduction of Cas9 coding sequence. The expression of Cas9 was confirmed by western blotting (Fig. S1a). Then we transduced MHCC97L-Cas9 with GeCKO v2A library which contains 65,386 unique sgRNA sequences targeting 19,052 human genes and 1864 miRNAs (3 sgRNAs per gene, 4 sgRNAs per miRNA, and 1000 non-targeting controls) at a low MOI (~0.3) to ensure effective barcoding of individual cells. Then, the transduced cells were selected with 1.6 μg/ml of puromycin for 7 days to generate a mutant cell pool, which was then treated with vehicle (DMSO) and Sorafenib (7 µM) for 7 days, respectively. After treatment, at least 3 × 10^7^ cells were collected for genomic DNA extraction to ensure over 400× coverage of GeCKO v2A library. The sgRNA sequences were amplified using NEBNext® High-Fidelity 2X PCR Master Mix and subjected to massive parallel amplicon sequencing carried out by Novogene Technology (Beijing, China). The sgRNA read count and hits calling were analyzed by MAGeCK v0.5.7 algorithm^42^.

### Transcriptome sequencing

Transcriptome sequencing (RNA-seq) were performed in MHCC97L cells treated with vehicle and Sorafenib in a different time frame. The library construction and massive parallel sequencing was performed by Novogene Technology. RNA-seq data quality was checked by FASTQC and analyzed by TopHat-Cufflinks pipeline.

### Establishment of PHGDH knockout and knockdown cell lines

The Cas9 stable-expressing MHCC97L cells (MHCC97L-Cas9) were established by lentiviral transduction of Cas9 coding sequence into the genome of cells. MHCC97L was a gift from Fudan University (Professor Z.Y. Tang, Shanghai, China). The expression of Cas9 protein was confirmed by a Cas9-specific antibody (Cell Signaling #65832, dilution 1:1000). The PHGDH knockout cell lines were established by overexpressing sgRNA targeting coding sequence of PHGDH near the ATG start codon in MHCC97L-Cas9 cells. After 48 h of viral transduction, the cells were selected by 2 μg/ml puromycin until uninfected cells were eliminated. The plasmids carrying Cas9 and sgRNA were lentiCas9-Blast (Addgene #52962) and lentiGuide-Puro (Addgene #52963) and were provided by Feng Zhang’s laboratory. The sgRNA sequences targeting PHGDH were either retrieved from GeCKO v2A library or designed by CRISPOR [3]. All the sgPHGDH sequences are listed as follows: sgPHGDH#2: 5′-tggacgaaggcgccctgctc-3′, sgPHGDH#3: 5′-ggctgcactggacgtgttta-3′, sgPHGDH#4: 5′-tggtggcagagcgaacaata-3′, sgPHGDH#12: 5′-tggtggcagagcgaacaata-3′, sgPHGDH#32: 5′-gatgacatcagcggtcacct-3′

For *PHGDH* stable knockdown model, MHCC-97L cells were infected with *PHGHD* targeting shRNA expressing plasmids (pLKO.1) lentiviral particles. The shRNA sequences used are listed as follows: shPHGDH#20: 5′–gcttcgatgaaggacggcaaa-3′, shPHGDH#32: 5′-cgcagaactcacttgtggaat-3′. *AKT1S1*, *TBL1Y*, and *SKAP2*, and *AMPD2* were established with the following shRNAs: shATK1S1: 5′–gcgacagattccttctattaa-3′, shTBL1Y: 5′–cgtcccaagtaataaagatgt-3′, shSKAP2: 5′–gctcctgataaacgtatatat-3′, and shAMPD2: 5′–gcgcacgtctatggatggcaa-3′. ATF4 knockdown cells were established with the following shRNA: shATF4-#73: 5′–gcctaggtctcttagatgatt-3′ and shATF4#75: 5′–gccaagcacttcaaacctcat-3′. *NRF2* stable knockdown cells were established as previously described^43,44^.

### qRT-PCR and western blotting

The mRNA expression of *PHGDH*, *PSAT1*, *PSPH*, and *HPRT* was determined by qRT-PCR with the primers: PHGDH-forward: 5′-acgtgtttacggaagagccg-3′, PHGDH-reverse: 5′-cccttcaccatgtccacgaa-3′; PSPH-forward: 5′-tgtcagaaatgacacggcga-3′, PSPH-reverse: 5′-gggggttgctctgctatgag-3′; PSAT1-forward: 5′-gaattgctagctgttccagaca-3′, PSAT1-reverse: 5′-tcagcacaccttcctgcttt-3′; HPRT-forward: 5′-cattatgctgaggatttggaaagg-3′ and HPRT-reverse: 5′- cttgagcacacagagggctaca-3′. Total RNA was extracted using TRIZOL. The cDNA was synthesized from 1 μg of total RNA by PrimeScript RT Master Mix (Takara Bio). The protein expression of PHGDH was determined by western blotting using Anti-PHGDH antibody produced in rabbit (HPA021241 SIGMA) at a dilution of 1:1000 using housekeeping gene α-tubulin antibody (#2144 Cell Signaling, dilution 1:1000) or monoclonal anti-β-Actin antibody (A5316 Sigma-Aldrich, dilution 1:1000) as a loading control. The Nrf2 protein expression was detected using polyclonal antibody (YT3189 Immunoway, 1:500). The total protein lysate was extracted by RIPA buffer.

### In vitro cell proliferation and apoptosis assay

In vitro cell proliferation rate was determined by monitoring the cell number for 7–9 days in cell culture. The cells in each condition were seeded at 3 × 10^4^ cells/well in a 12-well plate or 1 × 10^4^ cells/well in 24-well in triplicate and incubated in a 37 °C humidified CO_2_ incubator with the drug or vehicle containing medium refreshed every other day. The number of cells was determined by Z1 COULTER COUNTER® Cell and Particle Counter (Beckman Coulter). For the apoptosis assay, cells were seeded at 3 × 10^5^ cells/well into a six-well plate in triplicate. After 24 h, cells were subjected to starvation using 2% fetal bovine serum medium for 48 h and then followed by flow cytometry analysis. The cell apoptosis assay was determined according to the manual of FITC Annexin V Apoptosis Detection Kit I (BD Biosciences). Data were analyzed by FlowJo software (FlowJo).

### ROS measurement

To determine the intracellular ROS concentration, an equal number of HCC cells were seeded onto six-well dishes. Attached cells were trypsinized and washed with PBS. The cells were stained with 2 μmol/L oxidative stress indicator chloromethyl-2′,7′-dichlorodihydrofluorescein diacetate (CM-H2DCFDA) (Life Technologies, Austin, TX, USA) for analyzing ROS in the whole cell. Cells were stained with MitoSOX™ Red Mitochondrial Superoxide Indicator (Invitrogen™ M36008) for analyzing ROS level specifically in mitochondria. Stained cells were analyzed using the FACSCanto II Analyzer (BD Biosciences). The results were analyzed with FlowJo software (FlowJo).

### NADPH/NADP+ quantitation

Intracellular NADPH/NADP+ratios were quantitated using the NADP/NADPH Quantitation Colorimetric Kit (BioVision, Milpitas, CA, USA) according to the manufacturer’s manual. In brief, HCC cells were plated onto six-well dishes. Attached cells were trypsinized, washed with PBS, and extracted by two freeze/thaw cycles with the Extraction Buffer. A portion of extract underwent NADP decomposition by 60 °C heating for 30 min and another portion remained unheated (on ice). Both heated and unheated samples were subjected to NADP Cycling and were incubated in Cycling Buffer for 5 min followed by reaction development with the NADPH Developer. NADPH and NADP signal intensities were measured using a plate reader at OD of 450 nm. The NADPH/NADP+ ratio was calculated with the following equation: (Intensity of heated samples)/(intensity of unheated samples−intensity of heated samples).

### Metabolomics

To perform extraction of intracellular metabolites, plated MHCC97L-NTC and/or -shPHGDH cells were first washed with wash buffer which consisted of 5% (w/v) mannitol in H_2_O. After complete removal of the wash buffer, metabolite extraction was performed with methanol containing 10 μM of internal control solution (compound C1 with the *m*/*z* at 182.048 and compound A1 with the *m*/*z* at 231.070) (Human Metabolome Technologies, Tokyo, Japan). Extracted solutions underwent ultrafiltration by centrifugation through the centrifugal filter units (Human Metabolome Technologies). The filtered extracts were dried by centrifugal evaporation and the dried extracts were stored at −80 °C. CE-TOFMS was performed by Human Metabolome Technologies to analyze the extracted metabolites.

### C13 isotope tracing experiments

HCC cells were seeded at 5 × 10^5^ cells per dish in a 60-mm cell culture dish. After 24 h of seeding, cells were cultured in medium containing 25 mM [U-13C]-glucose and treated with 7 μm Sorafenib and vehicle respectively for 48 h and the intracellular metabolites were collected by three freeze–thaw cycle between liqN2 and 37 °C water bath in 50% MeOH. Serine quantification was carried out using LC-MS/MS-3200 Qtrap system. (Phase A: 0.1% formic acid in H_2_O, Phase B: 100% acetonitrile; serine Q1/Q3: 106/60). The intracellular serine content was analyzed by Analyst Software (SCIEX) and normalized to one million cells.

### Electron microscopy

MHCC97L cells were fixed with ice 4% formalin at 4oC. After replacement with 0.2 M sucrose, cells were fixed with 1% OsO4. Cells were rinsed and dehydrated with ethanol and placed in EMBed 812:proylene oxide in a desiccator. Cells were then embedded in Beam capsules and baked in oven at 60 °C oven and sectioned 0.5 µm thick and collected on grids. Grids were stained with uranyl acetate and then lead citrate for 5 min. Cells were imaged with a Philips CM100 transmission electron microscope.

### Animal experiments

Animal experiments in this study were carried out on male 6- to 8-week-old BALB/cAnN-nu (nude) mice. For orthotopic tumor implantation model, 1 × 10^6^ luciferase-labeled MHCC97L cells were firstly prepared and re-suspended in 100% Matrigel (BD Biosciences). The cells were injected into the left lobes of the livers of the nude mice. Tumor-bearing mice underwent bioluminescent imaging to examine tumor growth 6 weeks (42 days) post-implantation. The mice were administered 100 mg/kg d-luciferin (PerkinElmer, Waltham, MA, USA) via intraperitoneal injections and underwent imaging using the Xenogen IVIS 100 Imaging System (Caliper, Hopkinton, MA, USA). The livers and lungs of the mice were also harvested for ex vivo imaging. For the subcutaneous tumor injection model, 1 × 10^6^ MHCC97L cells were re-suspended in 50 μL PBS and 50 μL Matrigel (BD Biosciences) (1:1 ratio) before subcutaneous injections onto either flank of nude mice. For tumor measurement, an electronic caliper was used, and volumes were calculated using the formula as follows:

$${\mathrm{Tumor}}\,{\mathrm{volume}}\,\left( {{\mathrm{mm}}^{\mathrm{3}}} \right) = {\mathrm{length}}\,\left( {{\mathrm{mm}}} \right) \times {\mathrm{width}}\,\left( {{\mathrm{mm}}} \right) \times {\mathrm{height}}\,\left( {{\mathrm{mm}}} \right) \times 0.52.$$

Drug administration began 2 weeks post-injection where the tumors were deemed palpable. All experimental procedures on animals throughout the study were performed with prior approval obtained from the Committee on the Use of Live Animals in Teaching and Research of the University of Hong Kong. The experimental procedures were also performed according to the Animals (Control of Experiments) Ordinance of Hong Kong. All animal experiments were performed under the UK Co-ordinating Committee on Cancer Research (UKCCCR) Guidelines for the Welfare of Animals in Experimental Neoplasia^45^ to ensure minimal suffering of the animals throughout the procedures.

### Drug treatment

Sorafenib (Cat No.: S-8502), Regorafenib (Cat No.: R-8024), and Lenvatinib (Cat No.: L-5400) were purchased from LC Laboratories. Small molecular inhibitor NCT-503 (Cat No.: AOB9522) was purchased from AOBIOUS, and NCT502 (Cat No.: 19716) and CBR-5884 (Cat No.: 19236) were purchased from Cayman. *N*-acetyl-l-cysteine (NAC, Cat No.: A7250), α-ketoglutarate (α-KG, Cat No.: 75890), and *tert*-butyl hydroperoxide (tBHP, Cat No.: 458139) were purchased from Sigma-Aldrich. Hydrogen peroxide (H_2_O_2_, Cat No.: 386790) was purchased from Calbiochem. For the in vitro treatment, all the small molecular inhibitors were dissolved in DMSO as a vehicle. GI50 of each inhibitor was determined in MHCC97L cells by counting viable cells after 48 h of treatment. For gene expression assay_,_ cells were treated with 200 μM tBHP and 20 μM H_2_O_2_ for 24 h, respectively. The concentration for treatment of Regorafenib was 10 μM, and the concentration for treatment of Lenvatinib, NCT-502, NCT-503, and CBR-5884 was 40 μM, and the treatment time was 48 h. For rescue assay, cells were treated with α-KG at 4 mM and NAC at 5 mM for 48 h. For apoptosis assay to test the co-treatment effect, MHCC97L cells were cultured in serum reduced (2%) condition. For the in vivo treatment, the stock solution of Sorafenib was diluted in distilled water and was administrated orally every day at 30 mg/kg to 6–8 weeks BALB/cAnN-nu (nude) mice. The NCT-503 was dissolved in a vehicle containing 5% PEG400 and 5% Tween 80 and was administrated via intraperitoneal injection at 40 mg/kg every day. The treatment was conducted 7 days after tumor implantation.

### Reporting Summary

Further information on research design is available in the Nature Research Reporting Summary linked to this article.

## Supplementary information

Supplementary information

Reporting Summary

Source Data

## Data Availability

RNA-Sequencing data are available in NCBI BioProject (accession number PRJNA557663). The source data underlying Figs. 1a–c, 2a–d, 3a–g, 4a–g, 5a–d, 6a–d, and Supplementary Figs. 1a–e, 2a–d, 3a, b, 4a, b, 5a–d, 6a, b, and 7 are provided as a Source Data file. Publicly release data from Connectivity Map are available via GEO (accession GSE92742) and from the clue data library app. A reporting summary for this article is available as a supplementary information file.
