# Supplementary material for: Genome-wide CRISPR/Cas9 library screening identified PHGDH as a critical driver for Sorafenib resistance in HCC
Source: Nat Commun. 2019 Oct 15;10:4681. doi: 10.1038/s41467-019-12606-7 (PMC6794322; doi:10.1038/s41467-019-12606-7)
Supplement: Supplementary file 1 — Supplementary information [file 41467_2019_12606_MOESM1_ESM.pdf]

Supplementary information for the manuscript entitled:

Genome-wide CRISPR/Cas9 library screening identified PHGDH as a critical driver for Sorafenib resistance in HCC

Wei et al.

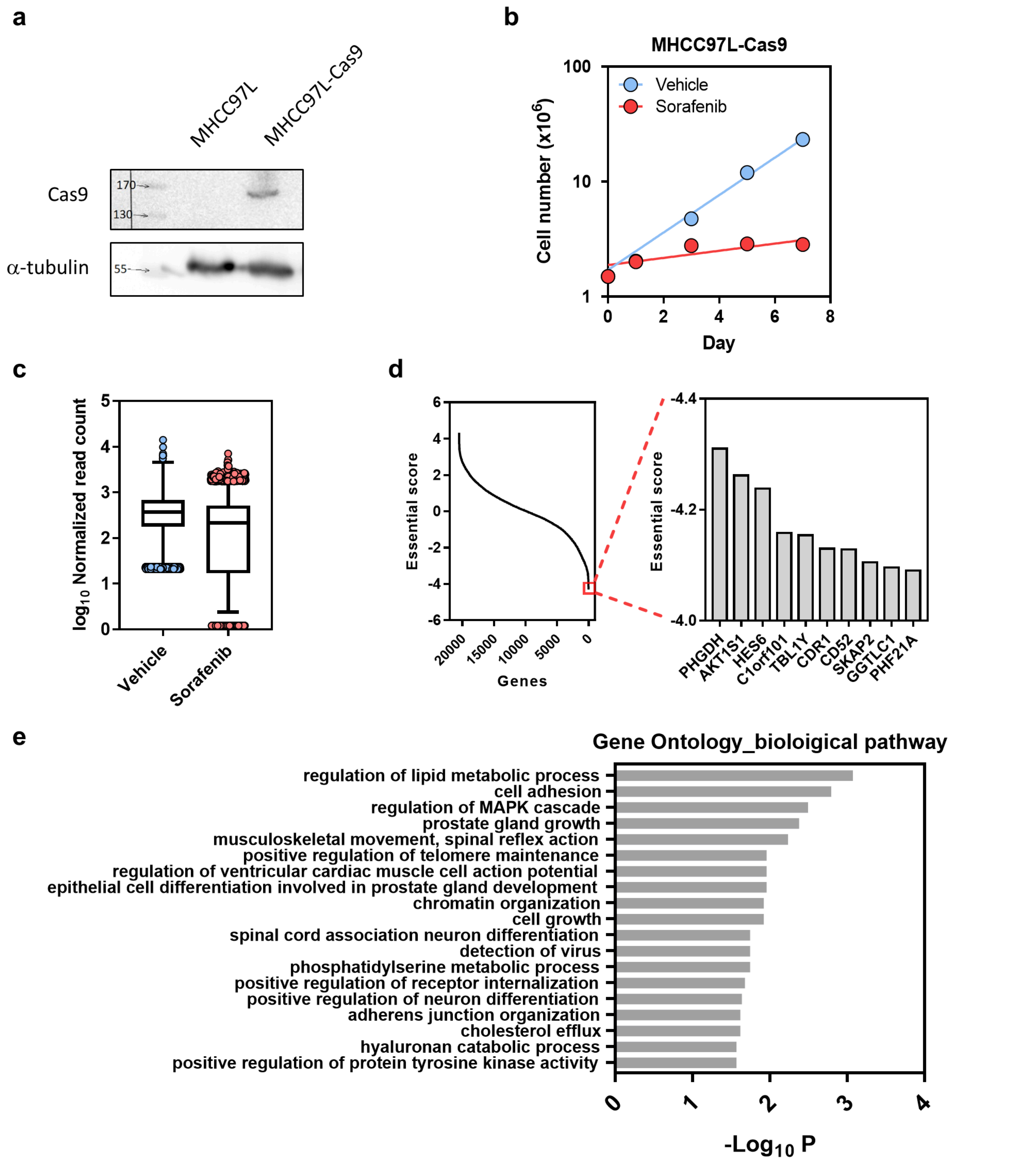

Supplementary Figure 1: CRISPR/Cas9 knockout library screening in HCC cells. **(a)** The stable expression of Cas9 was confirmed by Western blot. **(b)** The proliferation of HCC cell was significantly suppressed by Sorafenib treatment, thereby ensuring an effective selective pressure for the genetic screening (Blue connected dots: vehicle treated group; Red connected dots: Sorafenib treated group). **(c)** Comparison of the median read count of all sgRNAs in vehicle and Sorafenib treated samples. The overall reduced sgRNA median read count and more outliers in Sorafenib treatment suggested an effective library screening. **(d)** The top 10 negatively selected genes in the genome-wide CRISPR/Cas9 knockout screening. **(e)** Pathway analysis (DAVID Bioinformatics Resources 6.8) suggested that the negatively selected genes identified by genome-wide knockout screening in HCC cells upon Sorafenib treatment were involved in cell growth and adhesion, protein tyrosine kinase activity, lipid and phosphatidylserine metabolic process, and regulation of MAPK cascade. Source data are provided as a Source Data file. (Student t-test * $P < 0.05$, ** $P < 0.01$, *** $P < 0.001$)

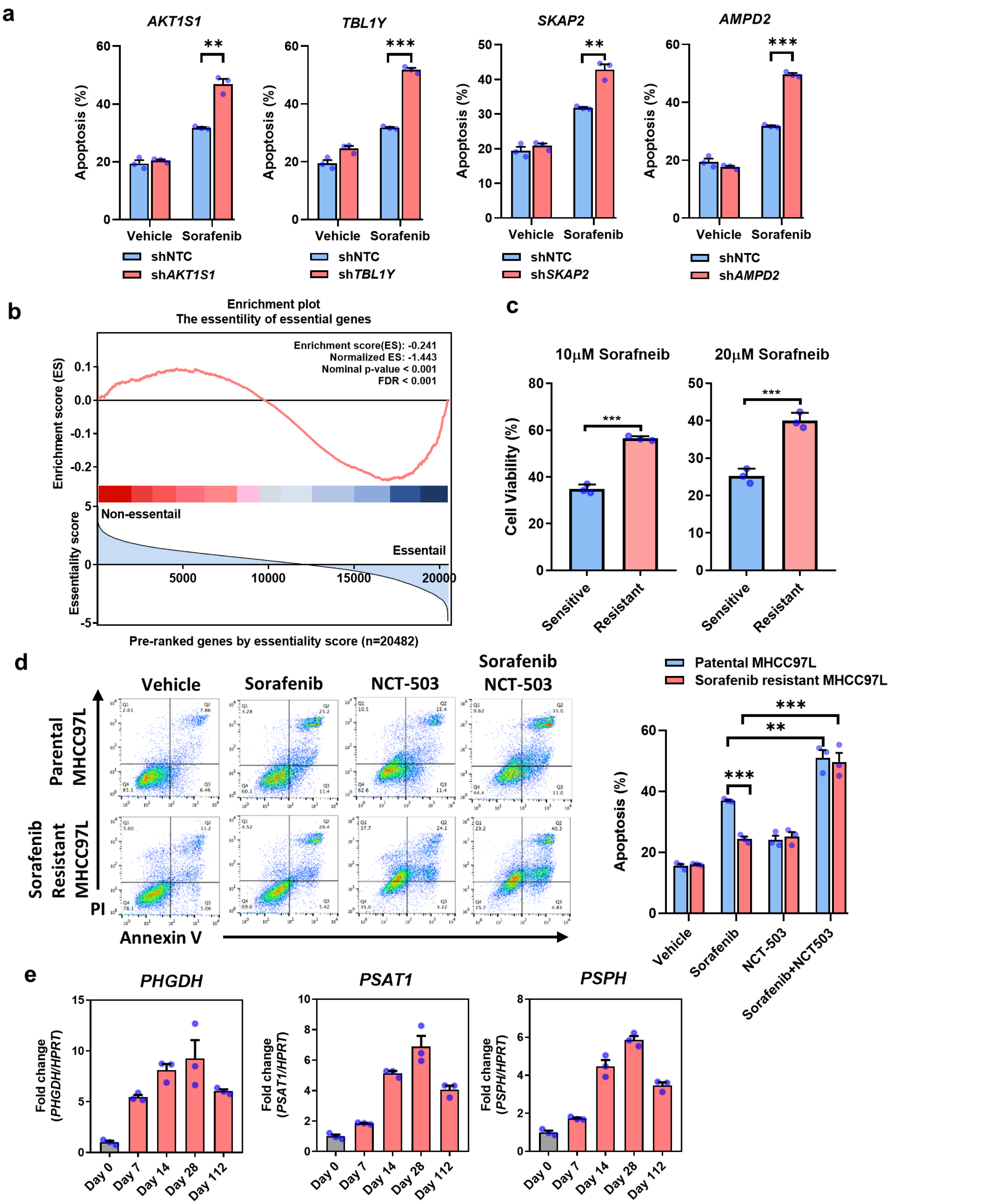

Supplementary Figure 2: Validation of the CRIPSR/Cas9 knockout library screening experiment. **(a)** Knockdown of the genes including AKT1S1, TBL1Y, SKAP2, and AMPD2 on the top of the candidate list also sensitized HCC cells to sorafenib treatment as suggested by the apoptosis assay (Blue bar: non-target control; Red bar: knockdown clones). **(b)** The previously reported 1580 essential genes were significantly depleted at day 7 of vehicle control group as compared to day 0. **(c)** The cell viability in Sorafenib resistant MHCC97L is higher than that of parental cells upon 10 mM and 20 mM sorafenib treatment. The apoptotic cell population was also dramatically decreased in resistant cells treated with 5uM Sorafenib. **(d)** Apoptosis of parental and Sorafenib resistant MHCC97L upon Sorafenib, NCT-503, and Sorafenib plus NCT-503 co-treatment were assessed by Annexin V/PI staining. Co-treatment of NCT-503 effectively overcame Sorafenib resistance in MHCC97L cells (Blue bar: MHCC97L parental cells; Red bar: Sorafenib resistant MHCC97L cells). **(e)** The induced mRNA expression of PHGDH, PSAT1, PSPH upon Sorafenib treatment was further validated by qRT-PCR. The error bar represents the SEM, n=3 biological independent samples. Source data are provided as a Source Data file. (Student t-test * $P < 0.05$, ** $P < 0.01$, *** $P < 0.001$)

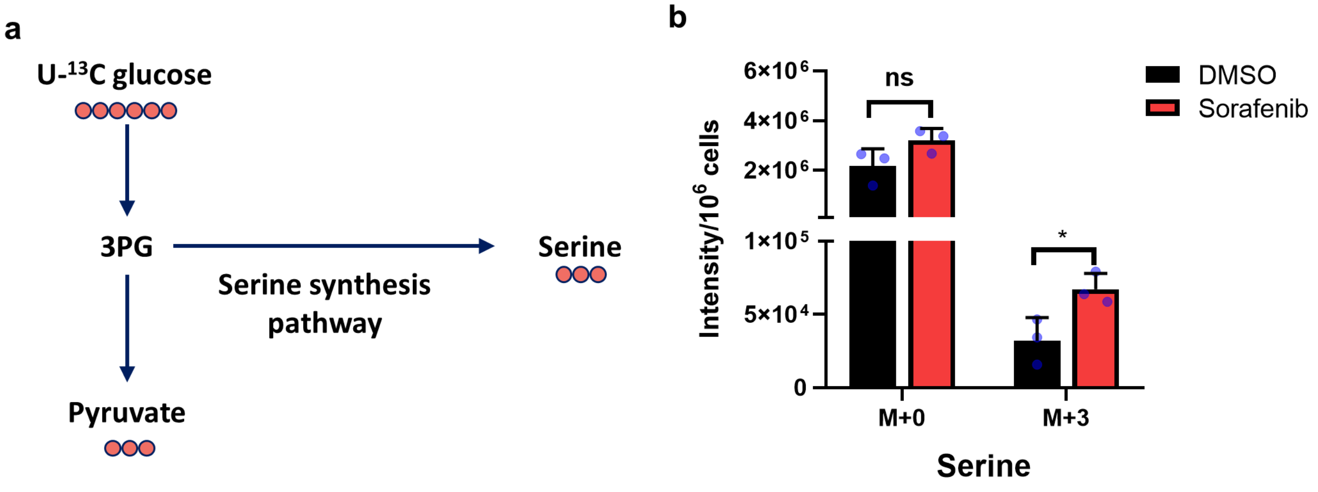

Supplementary Figure 3: The intracellular serine content was significantly induced by Sorafenib treatment. (a) The schematic diagram of C13 carbon tracing experiment in glycolytic metabolism and serine synthesis pathway. **(b)** The C13 serine in HCC cells was significantly increased upon Sorafenib treatment while the C12 serine was maintained in the same level (Black Bar: DMSO treated group; Red bar: Sorafenib treated group). The error bar represents the SEM, n=3 biological independent samples. Source data are provided as a Source Data file. (Student t-test * $P < 0.05$)

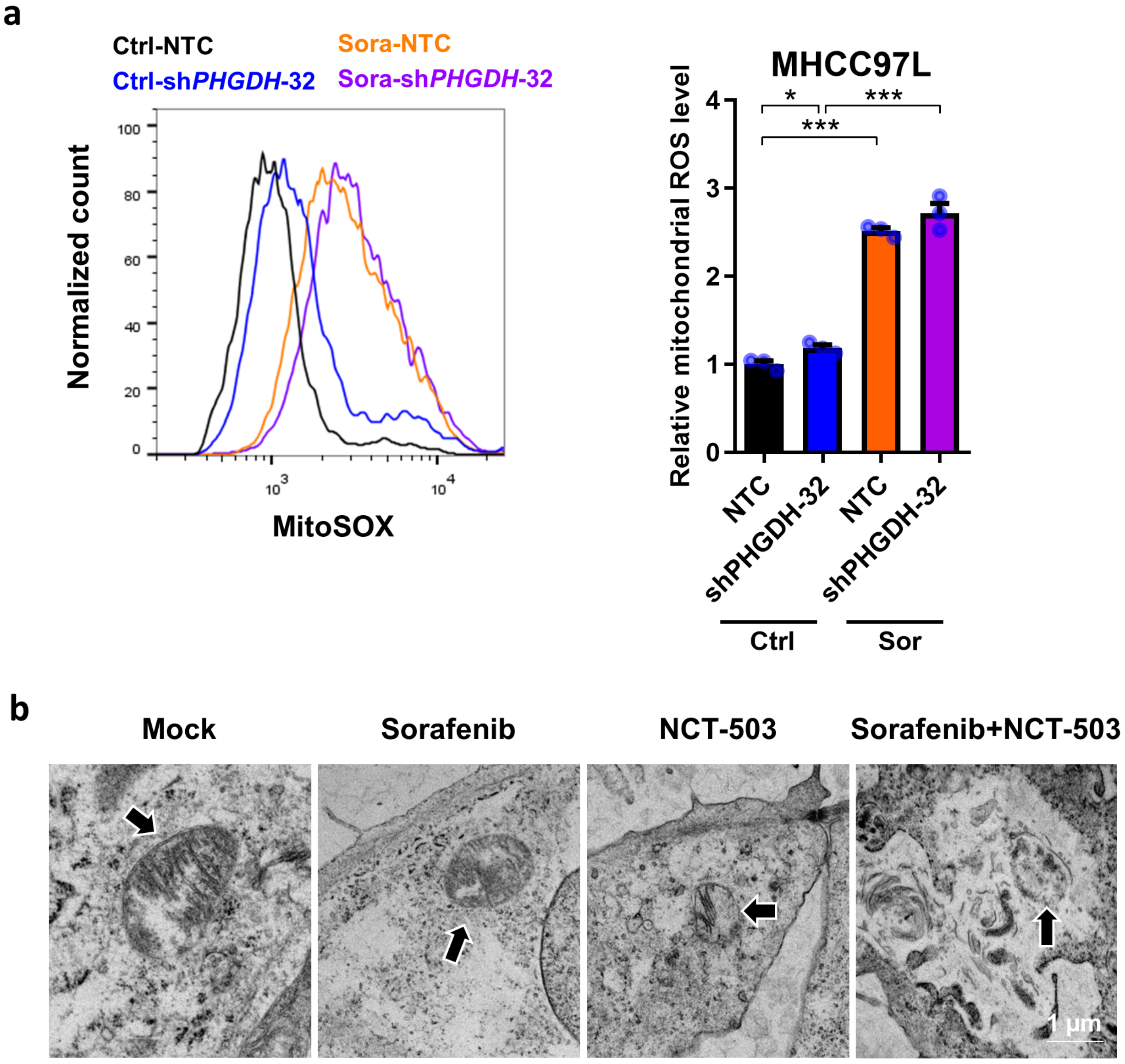

Supplementary Figure 4: The increased ROS in HCC cells upon Sorafenib treatment was produced in mitochondria. (a) Sorafenib treatment augmented ROS level in mitochondria of HCC cells. Knockdown of *PHGDH* intensified the Sorafenib-induced ROS in mitochondria of HCC cells (Black line: non-target control treated with vehicle; Orange line: non-target control treated with Sorafenib; Blue line: *PHGDH* knockdown clones treated with vehicle; Purple line: *PHGDH* knockdown clones treated with Sorafenib). **(b)** The cristae of mitochondria in HCC cells were less intact after single drug treatments and were further exacerbated with combined treatment. The error bar represents the SEM, n=3 biological independent samples. Source data are provided as a Source Data file. (Student t-test * $P < 0.05$, ** $P < 0.01$, *** $P < 0.001$)

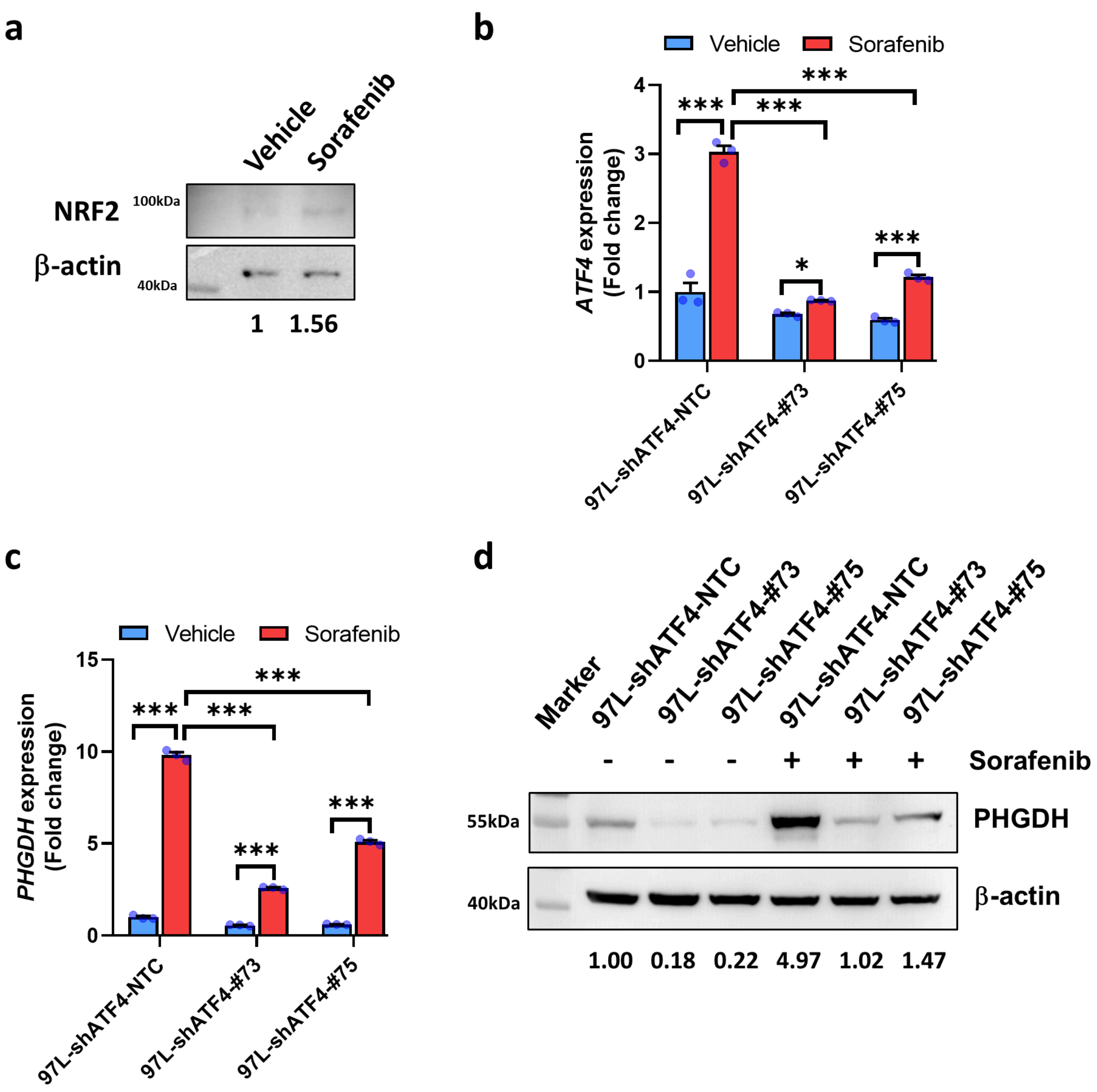

Supplementary Figure 5: *PHGDH* is one of the transcriptional targets of NRF2 and ATF4. (a) The expression of NRF2 was significantly induced in HCC cells treated with Sorafenib. **(b)** The mRNA expression of *ATF4* was successfully knocked down in HCC cells. Sorafenib treatment significantly induced *ATF4* expression in HCC cells but not that significant in *ATF4* knockdown cell lines (Blue bar: vehicle treated group; Red bar: Sorafenib treated group). **(c)** The expression of *PHGDH* upon Sorafenib treatment was significantly decreased in *ATF4* stable knockdown cells in mRNA level (Blue bar: vehicle treated group; Red bar: Sorafenib treated group). **(d)** The expression of PHGDH upon Sorafenib treatment was significantly decreased in *ATF4* stable knockdown cells in protein level. The error bar represents the SEM, n=3 biological independent samples. Source data are provided as a Source Data file. (Student t-test * $P < 0.05$, ** $P < 0.01$, *** $P < 0.001$)

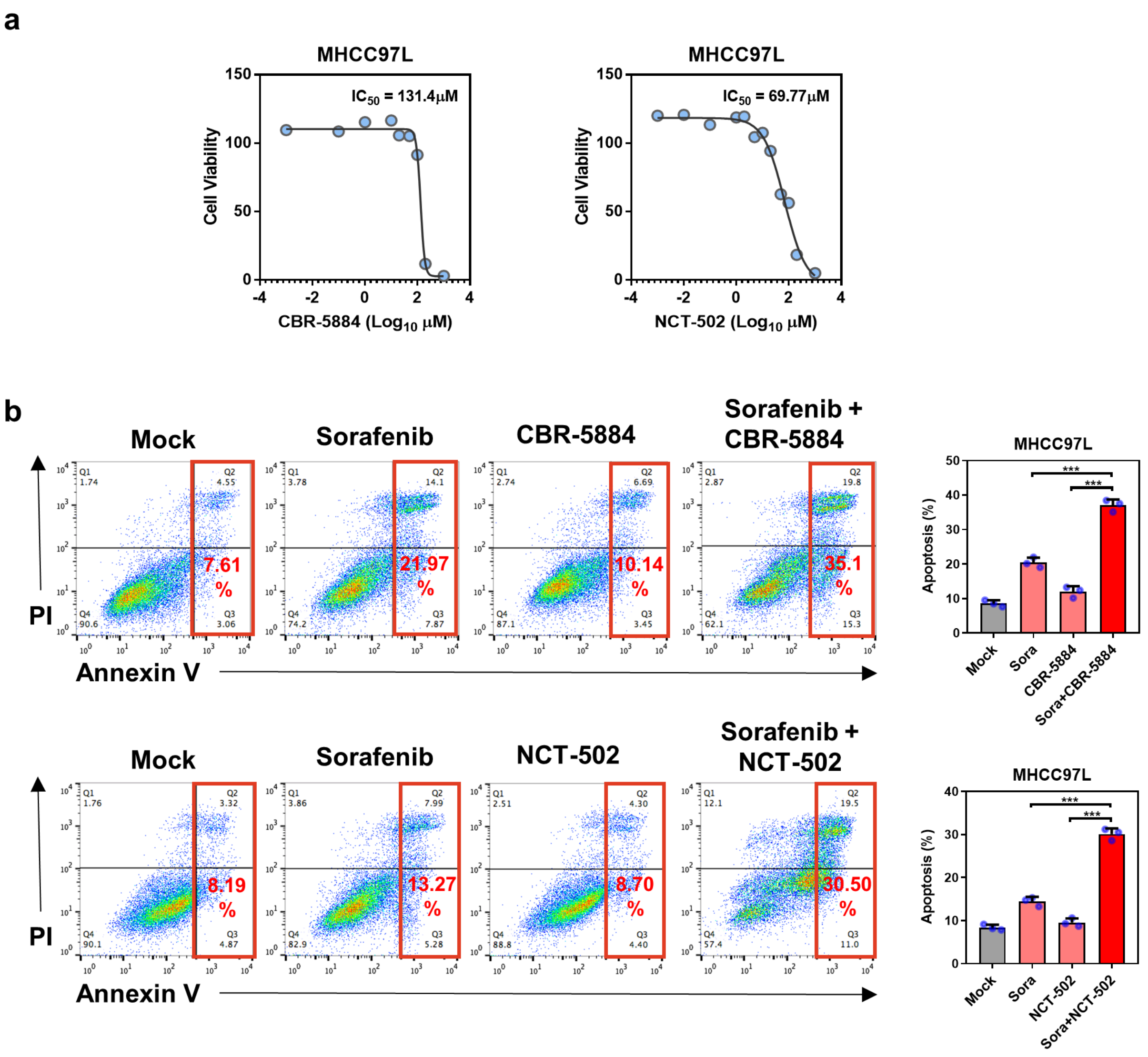

Supplementary Figure 6: PHGDH inhibitors, CBR-5884 and NCT-502, sensitized HCC cells to Sorafenib treatment. (a) The GI50 of CBR-5884 and NCT-502 were determined in MHCC97L cells. **(b)** Treatment of CBR-5884 and NCT-502 at 40μM for 48 hours could augment the Sorafenib induced apoptosis in HCC cells. The error bar represents the SEM, n=3 biological independent samples. Source data are provided as a Source Data file. (Student t-test * $P < 0.05$, ** $P < 0.01$, *** $P < 0.001$)

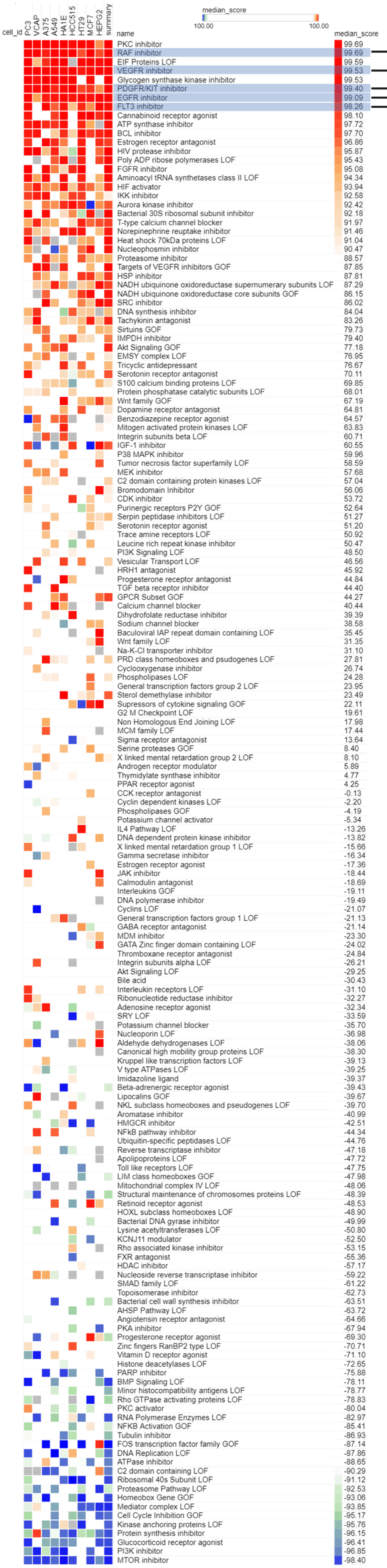

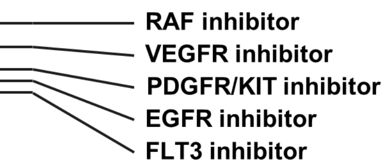

Supplementary Figure 7: SSP was induced in various cancers upon treatment of Sorafenib-like molecular. 171 pharmacology classes defined by CMap were ranked by their ability of inducing *PHGDH*, *PSAT1*, and *PSPH* expression across 9 common cell lines. Among them, the Sorafenib related pharmacology classes (RAF inhibitor, VEGFR inhibitor, PDGFR/KIT inhibitor, EGFR inhibitor, FLT3 inhibitor) are highly ranked in the list. Source data are provided as a Source Data file.

Supplementary Table 1: The summary of small molecules sharing the same mechanisms of actions with Sorafenib.

Drug	Mechanisms of action
vemurafenib	RAF inhibitor
PD-173074	VEGFR inhibitor
ENMD-2076	VEGFR inhibitor, FLT3 inhibitor
sorafenib	KIT inhibitor, RAF inhibitor, PDGFR receptor inhibitor, VEGFR inhibitor, FLT3 inhibitor, RET tyrosine kinase inhibitor
pazopanib	KIT inhibitor, PDGFR receptor inhibitor, VEGFR inhibitor
D-64406	PDGFR receptor inhibitor
PLX-4720	RAF inhibitor
sunitinib	KIT inhibitor, PDGFR receptor inhibitor, VEGFR inhibitor, FLT3 inhibitor, RET tyrosine kinase inhibitor
AG-879	VEGFR inhibitor
tivozanib	VEGFR inhibitor
HG-6-64-01	RAF inhibitor
dasatinib	KIT inhibitor, PDGFR receptor inhibitor
SU-1498	VEGFR inhibitor
tozasertib	FLT3 inhibitor
