# Supplementary material for: Genome-wide CRISPR/Cas9 library screening identified PHGDH as a critical driver for Sorafenib resistance in HCC
Source: Nat Commun. 2019 Oct 15;10:4681. doi: 10.1038/s41467-019-12606-7 (PMC6794322; doi:10.1038/s41467-019-12606-7)
Supplement: Supplementary file 2 — Reporting Summary [file 41467_2019_12606_MOESM2_ESM.pdf]

**目前的** PDF **瀏覽器不能顯示檔案的全部內容，請更新至最新的版本以便檢視文件。**
